# Generation of sphingosine-1-phosphate is enhanced in biliary tract cancer patients and is associated with lymphatic metastasis

**DOI:** 10.1038/s41598-018-29144-9

**Published:** 2018-07-17

**Authors:** Yuki Hirose, Masayuki Nagahashi, Eriko Katsuta, Kizuki Yuza, Kohei Miura, Jun Sakata, Takashi Kobayashi, Hiroshi Ichikawa, Yoshifumi Shimada, Hitoshi Kameyama, Kerry-Ann McDonald, Kazuaki Takabe, Toshifumi Wakai

**Affiliations:** 1 Division of Digestive and General Surgery, Niigata University Graduate School of Medical and Dental Sciences, Niigata City, Niigata, 951-8510 Japan; 2 Breast Surgery, Department of Surgical Oncology, Roswell Park Comprehensive Cancer Center, Buffalo, New York, 14263 USA; 3 Department of Surgery, University at Buffalo Jacobs School of Medicine and Biomedical Sciences, the State University of New York, Buffalo, New York, 14203 USA; 4 Department of Breast Surgery and Oncology, Tokyo Medical University, Shinjuku-ku, Tokyo, 160-8402 Japan; 5 Department of Surgery, Yokohama City University, Kanazawa-ku, Yokohama, 236-0004 Japan

## Abstract

Lymphatic metastasis is known to contribute to worse prognosis of biliary tract cancer (BTC). Recently, sphingosine-1-phosphate (S1P), a bioactive lipid mediator generated by sphingosine kinase 1 (SPHK1), has been shown to play an important role in lymphangiogenesis and lymph node metastasis in several types of cancer. However, the role of the lipid mediator in BTC has never been examined. Here we found that S1P is elevated in BTC with the activation of ceramide-synthetic pathways, suggesting that BTC utilizes SPHK1 to promote lymphatic metastasis. We found that S1P, sphingosine and ceramide precursors such as monohexosyl-ceramide and sphingomyelin, but not ceramide, were significantly increased in BTC compared to normal biliary tract tissue using LC-ESI-MS/MS. Utilizing The Cancer Genome Atlas cohort, we demonstrated that S1P in BTC is generated via *de novo* pathway and exported via ABCC1. Further, we found that SPHK1 expression positively correlated with factors related to lymphatic metastasis in BTC. Finally, immunohistochemical examination revealed that gallbladder cancer with lymph node metastasis had significantly higher expression of phospho-SPHK1 than that without. Taken together, our data suggest that S1P generated in BTC contributes to lymphatic metastasis.

## Introduction

Biliary tract cancer (BTC), the malignancy of the bile ducts and gallbladder, is a highly lethal disease in which a strong prognostic predictor is lymph node metastasis^1–5^. The incidence of lymph node metastasis ranges from 34 to 58%, and the prognosis of those with lymph node metastasis are very poor with the 5-year survival rates less than 30%^1–5^. In addition, our group has demonstrated that the main mode of intrahepatic invasion of gallbladder cancer is via lymphatic spread^6^. Thus, lymphatic invasion and lymph node metastasis determine the progression of BTC. Numerous studies have been conducted to elucidate the role of oncogenic proteins in lymph node metastasis; however, currently there is no effective target therapy clinically available for BTC.

Sphingosine-1-phosphate (S1P) is a lipid mediator generated inside the cells by sphingosine kinases (SPHK1 and SPHK2)^7–11^ and exported out of the cells through transporters^12–15^, which is known as “inside-out” signaling^12^. In breast cancer, we demonstrated that S1P generated by SPHK1 not only promoted angiogenesis, but also lymphangiogenesis, generation of new lymphatic vessels^16^. In agreement, we reported that patients with lymph node metastases had significantly higher expression of activated SPHK1 in breast and gastric cancer patients^17,18^. On the other hand, the role of the lipid mediator in BTC has been overlooked^19,20^. In the current study, we hypothesized that S1P plays a critical role in lymphangiogenesis, lymphatic invasion and lymph node metastasis in BTC.

S1P and its precursor sphingosine (Sph) are generated from ceramide. Interestingly, both ceramide and Sph induce apoptosis, whereas S1P promotes cell survival and growth. Thus the balance between ceramide and S1P is critical for cell fate, which is known as “sphingolipid rheostat”^21^. Recent advances in technology to determine the exact levels of sphingolipids, including S1P and ceramide both *in vitro* and *in vivo*, provides a better understanding of the role of S1P and ceramide in human patients and its potential mechanisms^22^. Thus, the aim of the current study was to elucidate the dynamics of S1P and ceramide, and its contribution to lymphangiogenesis, lymphatic invasion and lymph node metastasis in BTC.

## Results

### Activation of the sphingomyelin (SM) pathway generates ceramide in BTC compared with normal biliary tract tissue

**Figure 1:**
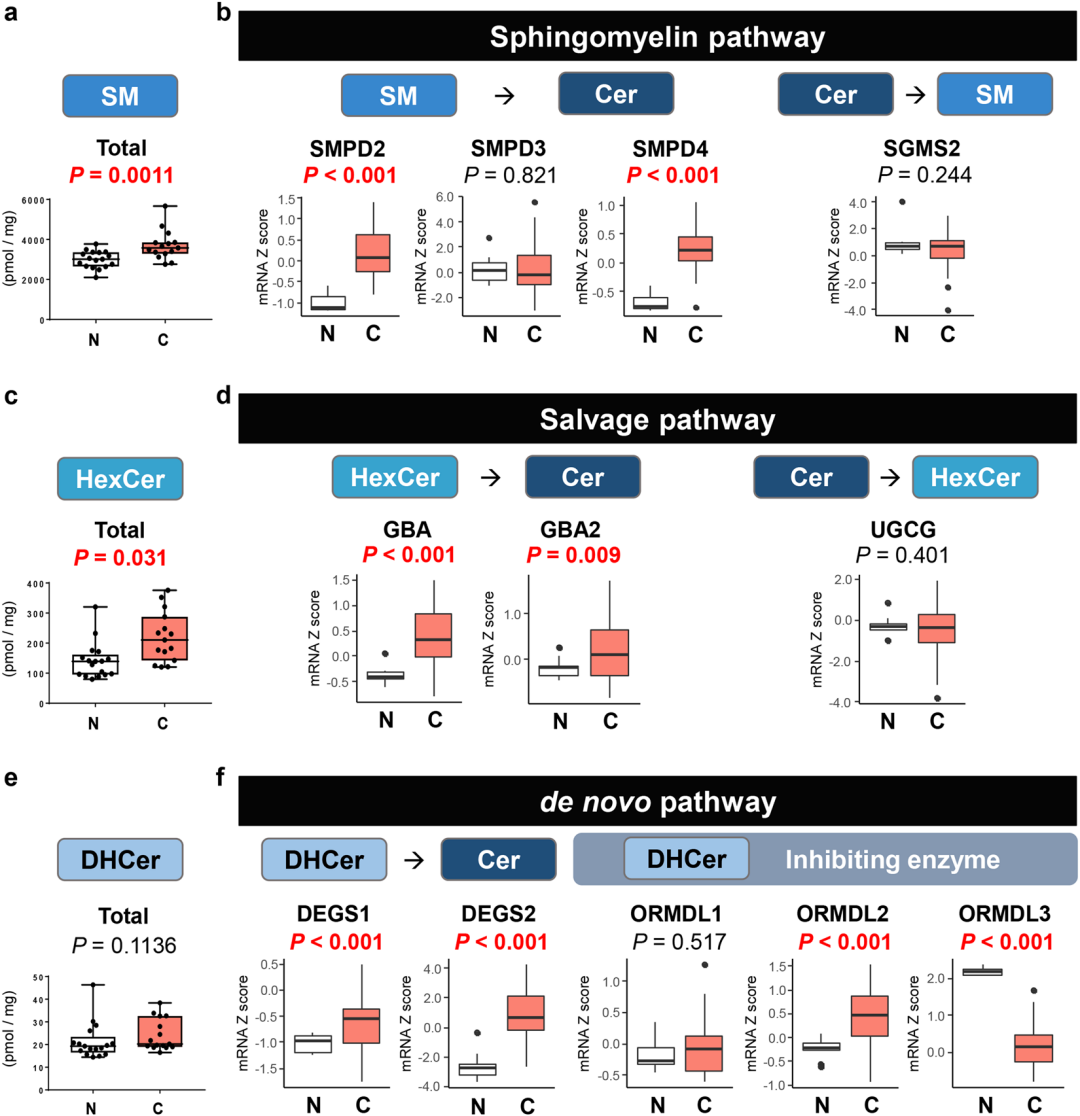
The major metabolic pathways for ceramide synthesis are enhanced in biliary tract cancer compared with normal biliary tract tissue. (**a**) Total SM levels in biliary tract cancer (n = 15) and normal biliary tract tissue (n = 17) were determined by LC-ESI-MS/MS. (**b**) The gene expression levels of SMPD2, SMPD3, SMPD4 and SGMS2 in TCGA cohort (36 and 9 samples of cancer and non-cancer tissue in cholangiocarcinoma cohort) were analyzed. (**c**) Total HexCer levels in biliary tract cancer (n = 15) and normal biliary tract tissue (n = 17) were determined by LC-ESI-MS/MS. (**d**) The gene expression levels of GBA, GBA2 and UGCG in TCGA cohort (36 and 9 samples of cancer and non-cancer tissue in cholangiocarcinoma cohort) were analyzed. (**e**) Total DHCer levels in biliary tract cancer (n = 15) and normal biliary tract tissue (n = 17) were determined by LC-ESI-MS/MS. (**f**) The gene expression levels of DEGS1, DEGS2, ORMDL1, ORMDL2 and ORMDL3 in TCGA cohort (36 and 9 samples of cancer and non-cancer tissue in cholangiocarcinoma cohort) were analyzed. Mean values are shown by the horizontal lines. Two-sided *P* values < 0.05 were considered statistically significant (Mann-Whitney U test). N: normal biliary tract tissue; C: biliary tract cancer tissue.

To clarify which ceramide synthesis pathway is responsible in BTC, we examined the SM pathway, which is one of major pathways to generate ceramide^22–25^. Total SM and most species of SM, except for C_14:0_- and C_18:0_-SM, were significantly higher in BTC than in normal biliary tract tissue (Fig. 1a and Supplementary Fig. 1).

The gene expression of SM phosphodiesterase (SMPD) 2 and SMPD4 that synthesize ceramide from SM were significantly higher in BTC than in normal tissue in The Cancer Genome Atlas (TCGA) cohort (Fig. 1b). Whereas the gene expression level of SM synthase 2 (SGMS2), which catalyzes ceramide to SM, was equivalent between cancer and normal tissue (Fig. 1b). This result indicates that the SM pathway is activated to produce ceramide in BTC tissue.

### Activation of the salvage pathway generates ceramide in BTC compared with normal biliary tract tissue

The salvage pathway, a major pathway of ceramide synthesis, was analyzed in BTC. Total monohexosylceramide (HexCer) and majority of HexCer species, which are antecedent to ceramide, were significantly higher in BTC than in normal tissue (Fig. 1c and Supplementary Fig. 2).

Interestingly, the gene expression of both glucosylceramidase beta (GBA) and GBA2, which encode a lysosomal membrane protein that produces ceramide from HexCer, were significantly higher in BTC than in normal tissue in TCGA cohort (Fig. 1d). On the other hand, there was no difference in the gene expression of ceramide glucosyltransferase (UGCG), which catalyzes ceramide back to HexCer (Fig. 1d). These data suggest that the salvage pathway is activated to promote ceramide synthesis in BTC tissue.

### Activation of the *de novo* pathway generates ceramide in BTC compared with normal biliary tract tissue

We investigated the *de novo* pathway, another major pathway for ceramide synthesis in the BTC. Contrary to SM or HexCer, there was no significant difference in the levels of total dihydro-ceramide (DHCer) and most species of DHCer, except for C_24:0_-DHCer, between BTC and normal tissue (Fig. 1e and Supplementary Fig. 3).

The gene expression of both DHCer desaturase (DEGS) 1 and DEGS2, which are enzymes that convert DHCer to ceramide, DHsph to Sph and DHS1P to S1P, were significantly higher in BTC than in normal tissue in TCGA cohort (Fig. 1f). Among ORMDL sphingolipid biosynthesis regulators (ORMDLs), which are a group of Orm family and regulate ceramide levels through inhibiting serine palmitoyltransferase, ORMDL2 was significantly higher and ORMDL3 was significantly lower in BTC than in normal tissue (Fig. 1f).

### Ceramide levels are not elevated in BTC compared with normal biliary tract tissue

**Figure 2:**
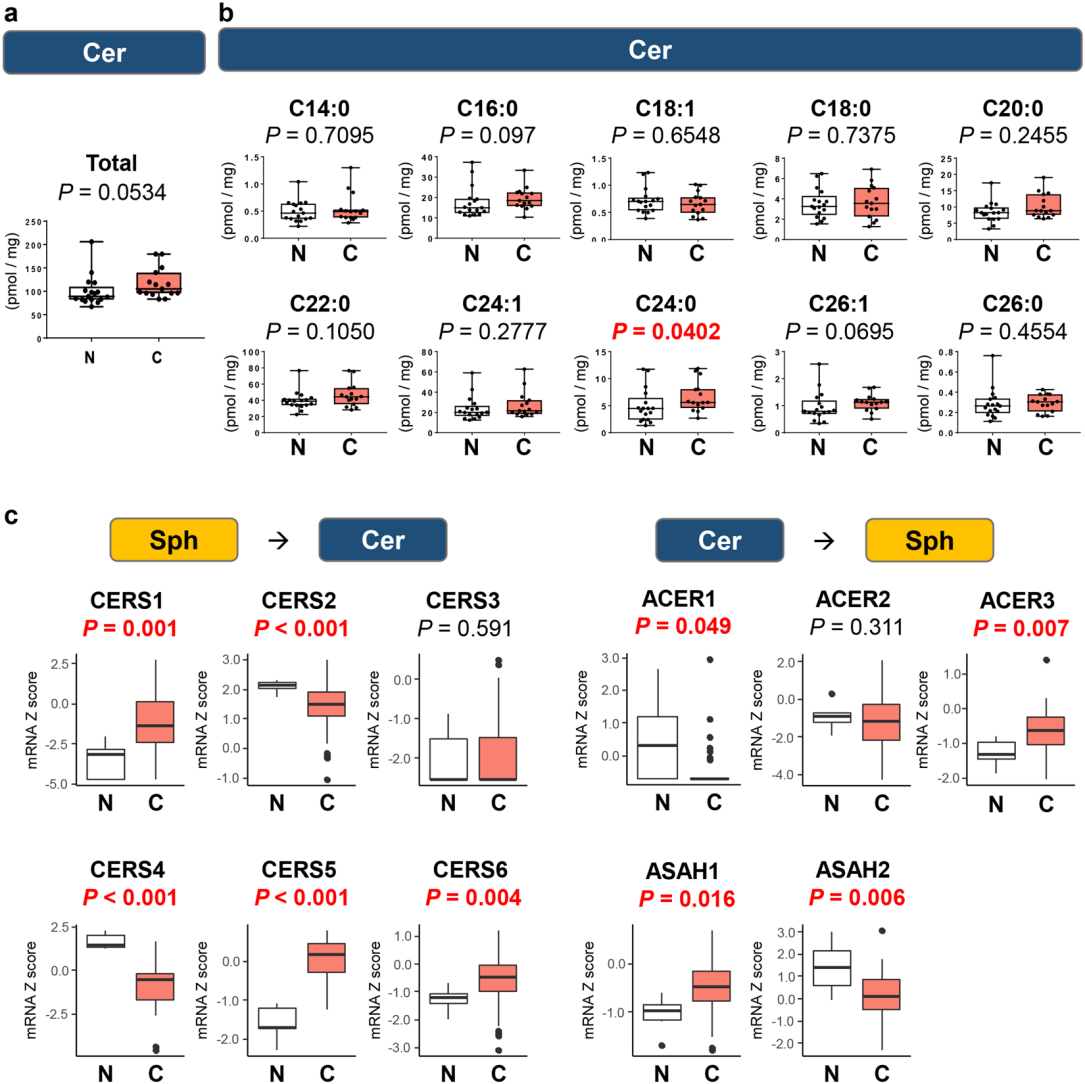
Ceramide is not increased in biliary tract cancer compared to normal biliary tract tissue. The levels of total ceramide (**a**) and each ceramide species (C14:0, C16:0, C18:1, C18:0, C20:0, C22:0, C24:1, C24:0, C26:1, and C26:0) (**b**) in biliary tract cancer (n = 15) and normal biliary tract tissue (n = 17) were determined by LC-ESI-MS/MS. (**c**) The gene expression levels of CERS1, CERS2, CERS3, CERS4, CERS5, CERS6, ACER1, ACER2, ACER3, ASAH1 and ASAH2 in TCGA cohort (36 and 9 samples of cancer and non-cancer tissue in cholangiocarcinoma cohort) were analyzed. Mean values are shown by the horizontal lines. Two-sided *P* values < 0.05 were considered statistically significant (Mann-Whitney U test). N: normal biliary tract tissue; C: biliary tract cancer tissue.

Ceramide is known to play a role in cancer cell growth arrest and apoptosis^21^. In contrast to elevated levels of its precursors (HexCer and SM) and expression of its synthase, there was no significant difference in the levels of total ceramide and the vast majority of ceramide species, except for C_24:0_-ceramide, between BTC and normal biliary tract tissue (Fig. 2a,b). Our results show that ceramide is generated, but not increased, in BTC compared with normal tissue. This suggests that it is further metabolized. Indeed, among enzymes that convert ceramide to Sph such as alkaline ceramidase (ACER) 1, ACER2, ACER3, N-acylsphingosine amidohydrolase (ASAH) 1 and ASAH2, the gene expression of ACER3 and ASAH1 were significantly higher in BTC than in normal tissue, while ACER1 and ASAH2 were significantly lower in TCGA cohort (Fig. 2c). Among enzymes that convert Sph to ceramide, the gene expression of ceramide synthase (CER) 1, CER5 and CER6 were significantly higher in BTC than in normal tissue, while CERS2 and CERS4 were significantly lower in BTC tissue in TCGA cohort (Fig. 2c).

Ceramide-1-phosphate (C1P) promotes cell survival and cell migration. In the current study, the level of C1P was not measured due to technical difficulty. The gene expression of ceramide kinase (CERK) that converts ceramide to C1P was significantly higher in BTC than in normal tissue, which indicates that there is more C1P in BTC than in normal tissue. In contrast, the gene expression of phospholipid phosphatases (PLPPs), which converts C1P back to ceramide, was also determined. While expression of PLPP3 in BTC was significantly higher than that in normal tissue, expression of PLPP2 in BTC was significantly lower than that in normal tissue (Supplementary Fig. 4).

### S1P levels as well as gene expression of S1P generating enzymes are elevated in BTC compared with normal biliary tract tissue

**Figure 3:**
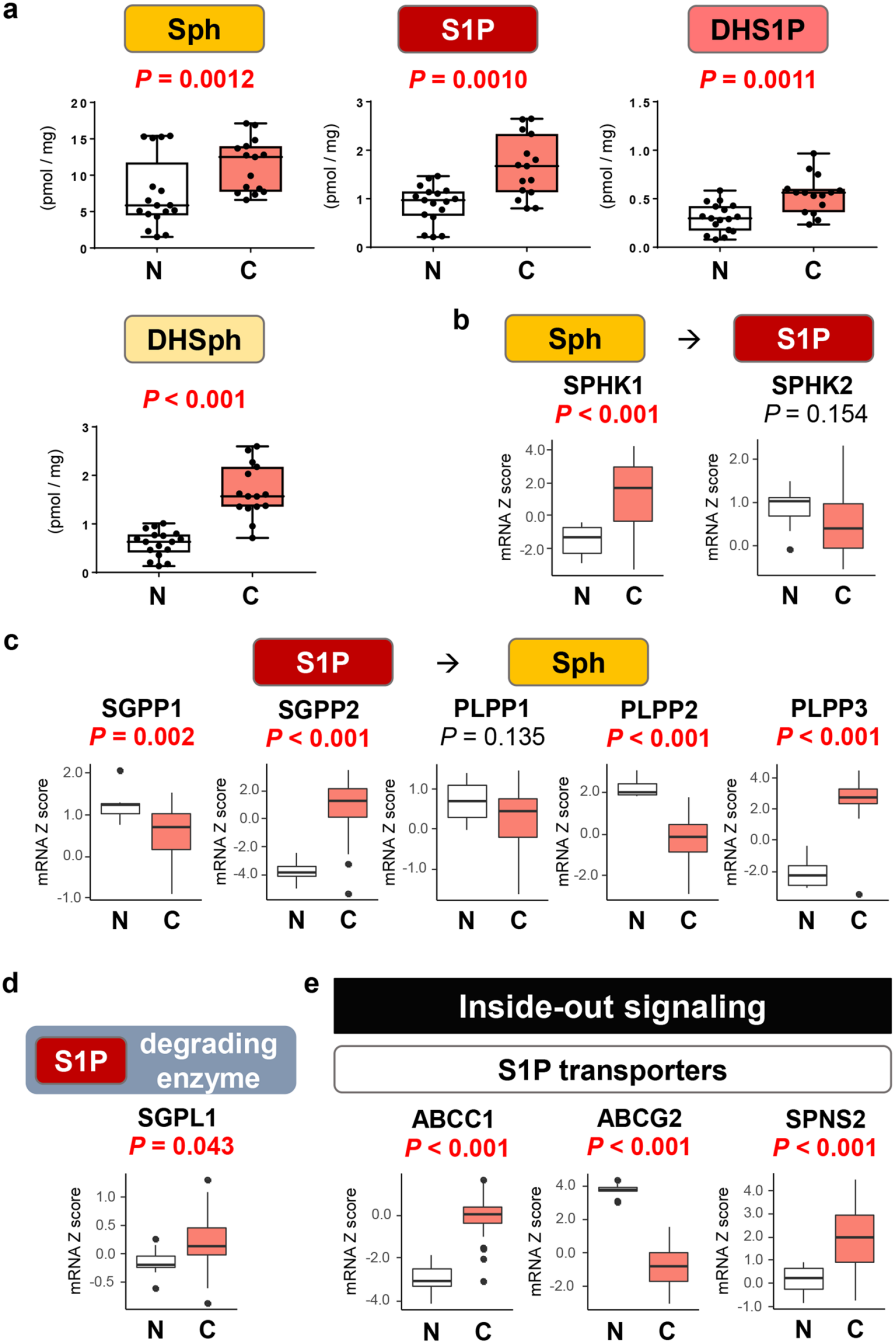
The levels of S1P and SPHK1 are increased in biliary tract cancer compared with normal biliary tract tissue. (**a**) The levels of the S1P, DHS1P, Sph and DHSph were determined by LC-ESI-MS/MS. (**b**) The gene expression levels of SPHK1 and SPHK2 in TCGA cohort (36 and 9 samples of cancer and non-cancer tissue in cholangiocarcinoma cohort) were analyzed. (**c**) The gene expression levels of SGPP1, SGPP2, PLPP1, PLPP2 and PLPP3 in the TCGA cohort were analyzed. (**d**) The gene expression level of SGPL1 in the TCGA cohort was analyzed. (**e**) The gene expression levels of ABCC1, ABCG2 and SPNS2 in the TCGA cohort were analyzed. Mean values are shown by the horizontal lines. Two-sided *P* values < 0.05 were considered statistically significant (Mann-Whitney U test). N: normal biliary tract tissue; C: biliary tract cancer tissue.

The levels of S1P, DHS1P, Sph and dihydro-Sph (DHSph) were found to be elevated in BTC compared with normal biliary tract tissue (Fig. 3a). S1P is generated from Sph by SPHK1 or sphingosine kinase 2 (SPHK2), and is converted back to Sph by S1P phosphatase 1 (SGPP1), SGPP2, PLPP1, PLPP2 or PLPP3 or degraded by S1P lyase 1 (SGPL1). It has been reported that main enzyme that produces S1P is SPHK1, not SPHK2 in cancer cells. Indeed, we have observed that the gene expression of SPHK1 was significantly higher in BTC than in normal tissue, while that of SPHK2 was not elevated in TCGA cohort (Fig. 3b). On the other hand, the gene expression of S1P-degrading enzymes varies; SGPP2, PLPP3 and SGPL1 were significantly higher in BTC than in normal tissue, while SGPP1 and PLPP2 were significantly lower in BTC tissue (Fig. 3c,d).

S1P is exported out of cells by three main transporters, ABCC1, ABCG2 and protein spinster homolog 2 (SPNS2), which is known as “inside-out signaling”. The gene expression of ABCC1 and SPNS2 were significantly higher in BTC than in normal tissue, while that of ABCG2 was lower in BTC tissue (Fig. 3e). These findings suggest a possibility that ABCC1 and SPNS2 are major transporters in BTC.

### SPHK1 expression positively correlates with the expression of lymphatic metastasis-related genes in BTC

**Figure 4:**
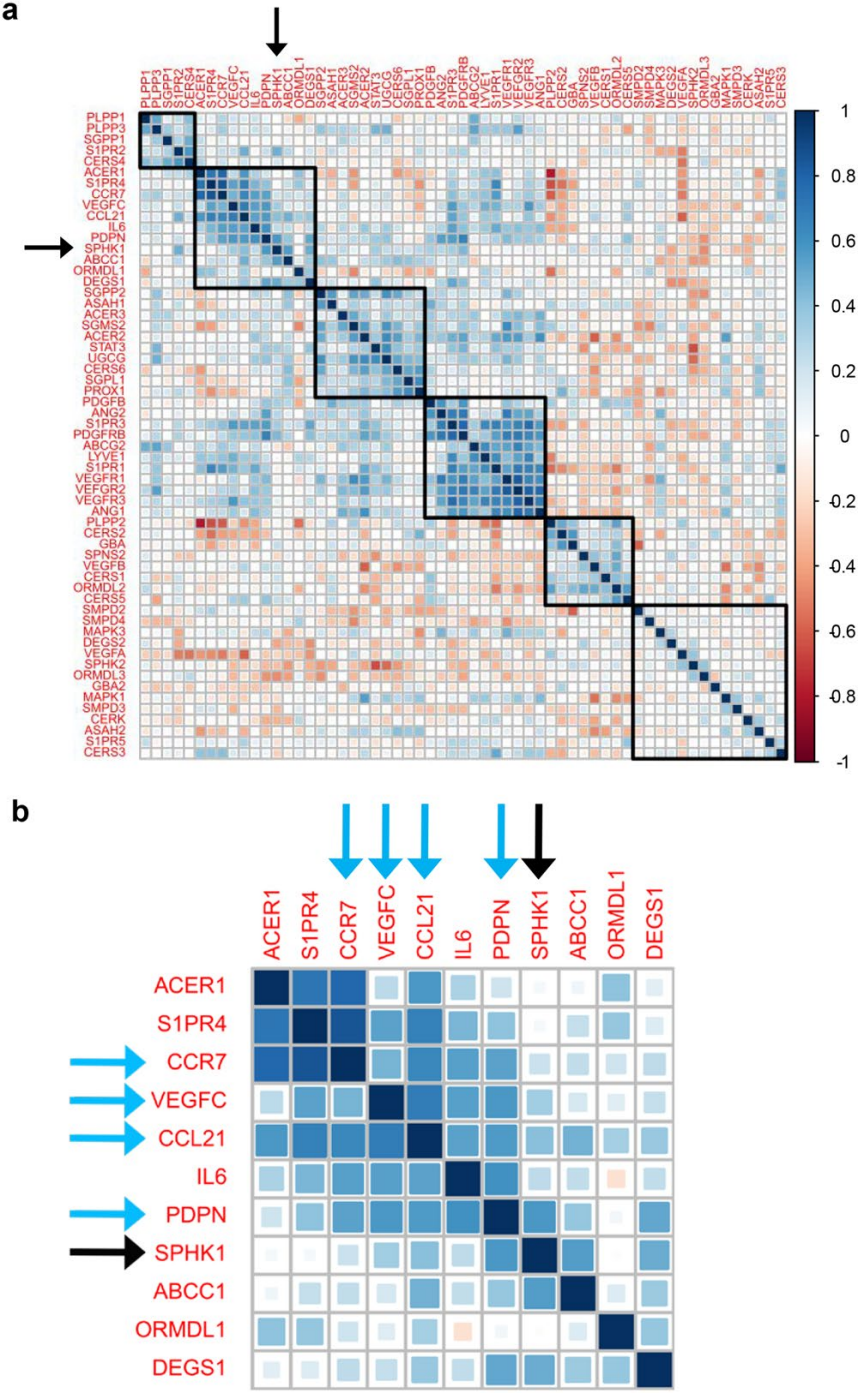
The gene expression level of SPHK1 is associated with those of lymphangiogenesis-related or lymphatic metastasis-related substrates in biliary tract cancer. (**a**) The correlation matrix analysis was performed for gene expression levels of substrates associated with S1P and ceramide biosynthetic pathway, angiogenesis, lymphangiogenesis or lymphatic metastasis in biliary tract cancer in TCGA cohort (36 samples of cancer tissue in cholangiocarcinoma cohort). (**b**) Substrates in the same group with SPHK1 in the correlation matrix analysis were closed up. Pearson correlations were calculated based on the expression levels of interested genes in cancer tissues. The hierarchical clustering method was used for the grouping of the genes. Blue squares indicated positive correlation and red squares indicated inverse correlation. Black arrows pointed to SPHK1 and blue arrows pointed to lymphangiogenesis-related or lymphatic metastasis-related substrates.

**Figure 5:**
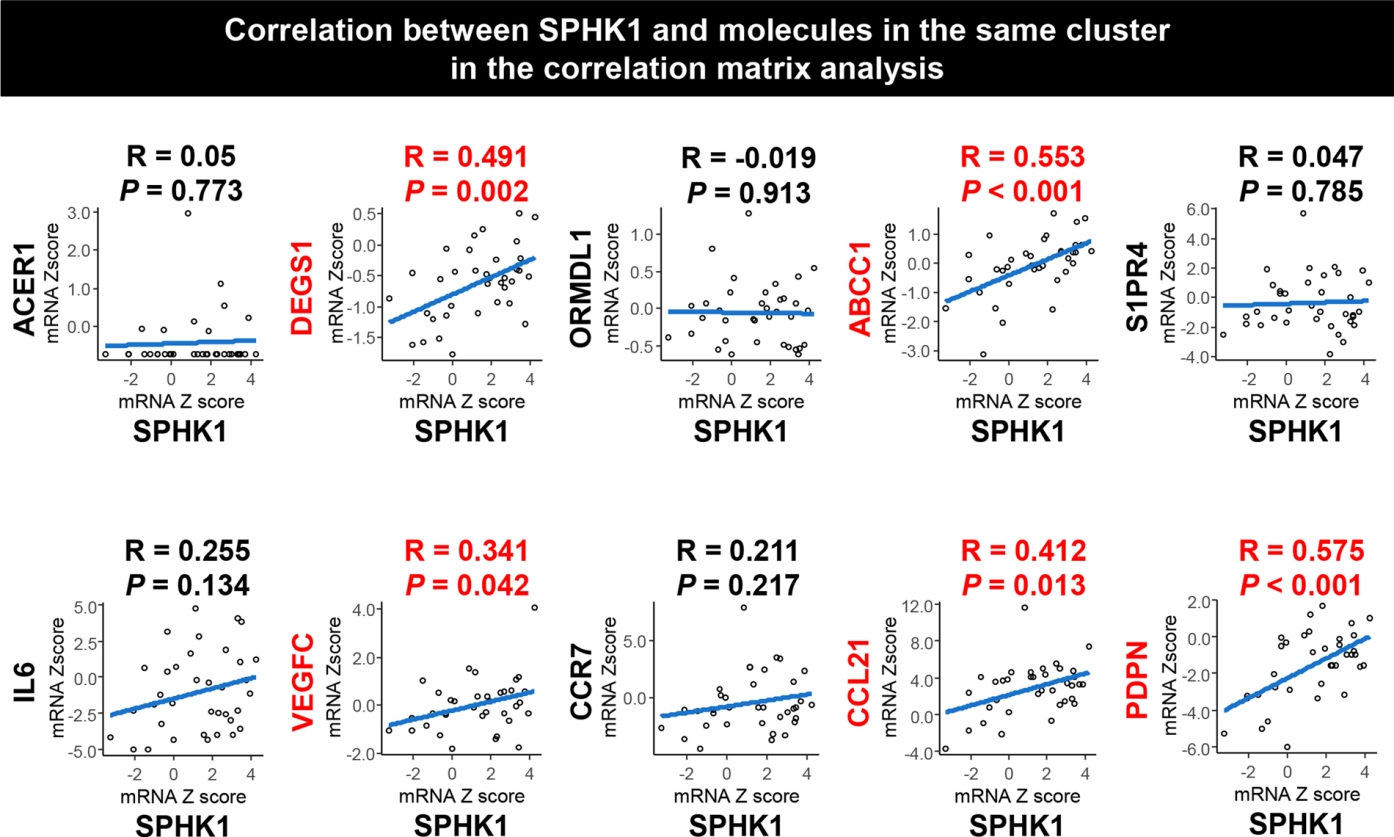
The gene expression level of SPHK1 positively correlated with those of lymphangiogenesis-related or lymphatic metastasis-related substrates in biliary tract cancer. Pearson correlations were calculated between gene expression level of SPHK1 and those of ACER1, DEGS1, ORMDL1, ABCC1, S1PR4, IL6, VEGFC, CCR7, CCL21 and PDPN in biliary tract cancer in TCGA cohort (36 samples of cancer tissue in cholangiocarcinoma cohort). All tests were two-sided, and *P* values < 0.05 were considered statistically significant.

We investigated the correlation of the gene expression levels among factors and enzymes associated with S1P and ceramide biosynthetic pathways and signaling in BTC using correlation matrix analysis in TCGA cohort (Fig. 4a). We also included factors related to angiogenesis, lymphangiogenesis, or lymphatic metastasis in the analysis because S1P is known to be involved in those processes^16,17^. SPHK1 was found in the same cluster with ABCC1, ACER1, C-C motif chemokine receptor 7 (CCR7), C-C motif chemokine ligand 21 (CCL21), DEGS1, interleukin 6 (IL6), ORMDL1, podoplanin (PDPN), S1P receptor 4 (S1PR4) and vascular endothelial growth factor C (VEGFC) (Fig. 4a,b). We then analyzed the correlation between the gene expression of SPHK1 and that of substrates in the same cluster with SPHK1 (Figs 4b, 5). In addition, we examined the correlation between the gene expression of SPHK1 and that of other lymphangiogenesis-related or lymphatic metastasis-related factors such as PROX1, LYVE1, PDGFB, and PDGFRβ (Supplementary Fig. 5). The gene expression of SPHK1 positively correlated with that of ABCC1, CCL21, DEGS1, PDPN, VEGFC, PDGFB, and PDGFRβ (Fig. 5 and Supplementary Fig. 5), while not with LYVE1 and PROX1. CCL21, PDPN, VEGFC, PDGFB, and PDGFRβ are well known for their roles in lymphatic metastasis^26–38^, whereas LYVE1 and PROX1 are known as lymphangiogenesis-related factors^36^. These results suggest that the gene expression of SPHK1, the main enzyme for S1P synthesis, positively correlated with expression of DEGS1 among all the enzymes involved in generation of S1P, and expression of ABCC1, the transporter of S1P. Further, SPHK1 expression correlated with expression of lymphatic metastasis-related factors, but not with angiogenesis-related or lymphangiogenesis-related factors in BTC.

SPHK2 is the other main enzyme that produces S1P. Thus, we analyzed the correlation between the gene expression of SPHK2 and lymphangiogenesis-related or lymphatic metastasis-related factors (Supplementary Fig. 6). As a result, there was no positive correlation between them.

### Phospho-SPHK1 is overexpressed in BTC with lymph node metastasis

**Figure 6:**
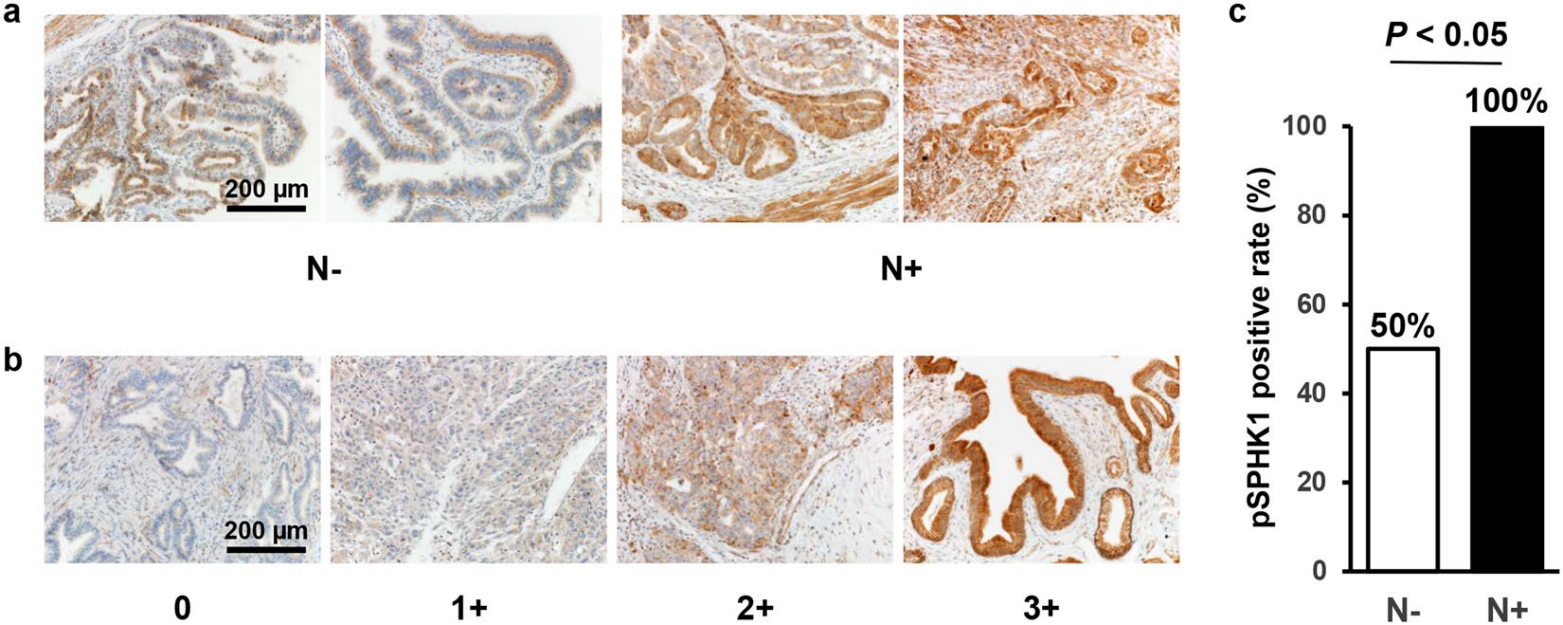
pSPHK1 is overexpressed in biliary tract cancer with lymph node metastasis on immunohistochemistry. (**a**) Semi-quantitative analysis of the immunohistochemical pSPHK1 expression in gallbladder cancer tissues with (n = 10) or without lymph node metastasis (n = 10) was performed. A staining score was calculated by multiplying two scores of staining intensity (0: negative staining; 1: weak staining; 2: moderate staining; 3: strong staining) and extent (0: 0–25%; 1: 26–50%; 2: 51–75%; 3: 76% -) of biliary tract cancer cells staining positive. The score of 0 to 5 was considered pSPHK1-negative, and 6 to 12 pSPHK1-positive. The score was compared between gallbladder cancer tissues with (n = 10) or without lymph node metastasis (n = 10) using Fisher’s exact test. The test was two-sided, and *P* value < 0.05 was considered statistically significant. Scale bar = 200 µm. (**b**) The staining intensity was determined and scored as 0 (negative staining), 1 (weak staining), 2 (moderate staining) and 3 (strong staining). Scale bar = 200 µm. (**c**) The result of immunohistochemical staining of pSPHK1 for gallbladder cancer with or without lymph node metastasis is shown.

SPHK1 is activated by the phosphorylation of Ser-225 (pSPHK1), which converts Sph to S1P^17,18^. We examined the pSPHK1 expression levels in BTC with or without lymph node metastasis by immunohistochemistry (Fig. 6a). pSPHK1 was expressed throughout the cytoplasm of the cancer cells, the intensity of which was scored as shown (Fig. 6b). pSPHK1 expression was significantly higher in BTC with lymph node metastasis than without (Fig. 6c). In addition, we evaluated the association between the pSPHK1 expression and lymphatic vessel density (LVD) in BTC, which showed no association between them (Supplementary Fig. 7a,b). These results suggest that pSPHK1 is not associated with lymphangiogenesis, but is associated with lymph node metastasis in BTC.

## Discussion

**Figure 7:**
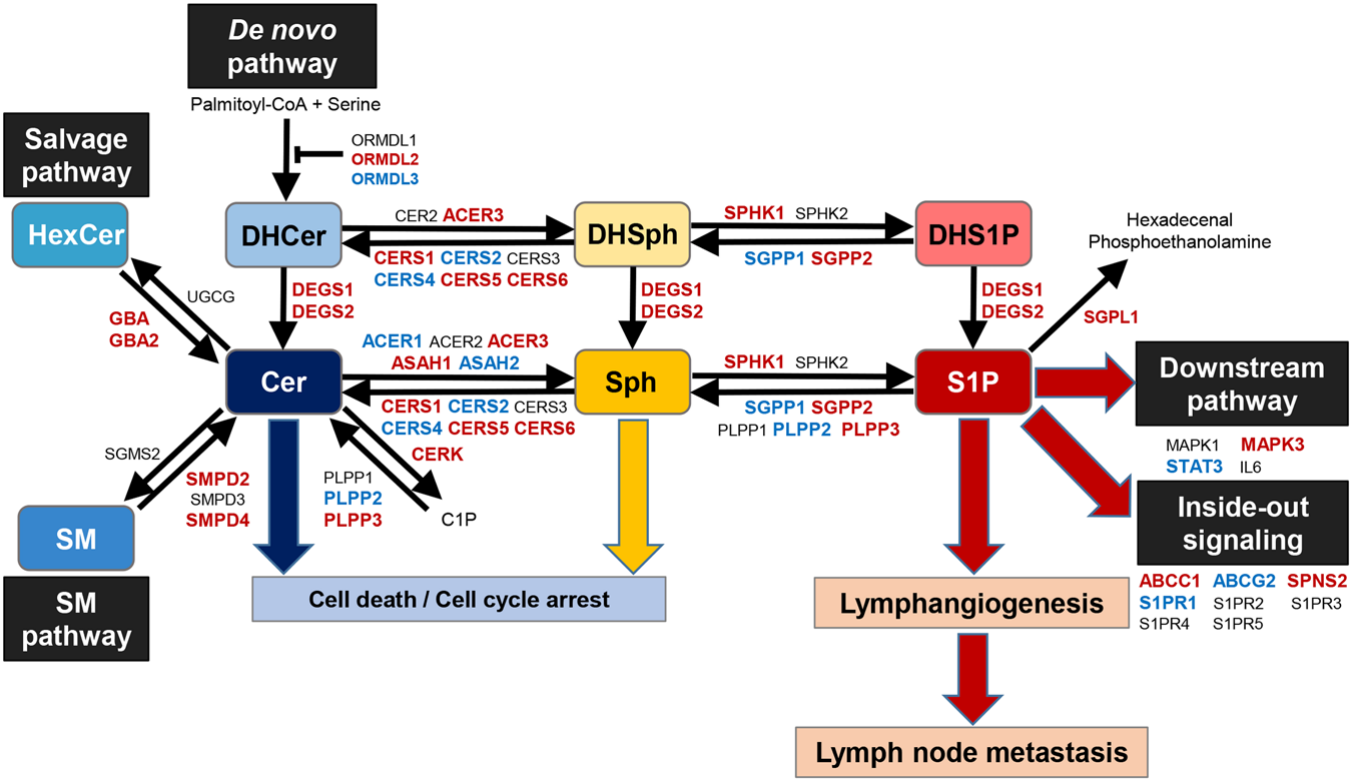
The dynamics of S1P- and ceramide-synthesis pathway. The gene expression levels of substrates with red capitals were increased in biliary tract cancer compared to normal biliary tract tissue, those with blue capitals were decreased in biliary tract cancer compared to normal biliary tract tissue, and those with black capitals were equivalent between them according to TCGA cohort.

Recent advances in lipidomics using mass spectrometry allow us to determine exact levels of sphingolipids including S1P, Sph, SM, HexCer, and ceramide. To our knowledge, this is the first study to quantify the levels of sphingolipids, including S1P and ceramide, in human BTC and normal biliary tract tissue. We found that S1P levels are elevated in BTC, where expression of SPHK1, the main enzyme that generates S1P, positively correlated with the expression of the DEGS1 enzyme and the ABCC1 transporter (Fig. 7). Further, SPHK1 expression positively correlated with lymphatic metastasis-related factors, and activated SPHK1 associated with lymph node metastasis in BTC.

In the current study, SM and HexCer, which are precursors of ceramide in the SM and salvage pathways respectively, were increased in BTC, whereas DHCer, the precursor in the *de novo* pathway, was not. Most of the enzymes converting ceramide precursors to ceramide increased in BTC, which indicate that ceramide synthesis is activated in BTC. Among all the enzymes that are involved in generation of S1P, only DEGS1 which catalyzes DHCer to Ceramide positively correlated with SPHK1, the major S1P synthase. This result suggests that BTC utilizes the *de novo* pathway and DEGS1 for generation of S1P. Furthermore, our results show that ABCC1 correlated with SPHK1 expression and suggests that S1P is exported by ABCC1 to the tumor microenvironment.

Previous studies have demonstrated that S1P and SPHK1 are associated with lymphatic metastasis in various types of cancer including breast, gastric, colon, liver and ovarian cancer^16,17,39–45^. These reports led us to hypothesize that SPHK1 is associated with lymphatic metastasis of BTC. In the current study, in BTC, the gene expression of SPHK1 positively correlated with VEGFC, PDPN, CCL21, PDGFB and PDGFRβ, which are all lymphatic metastasis-related factors. VEGFC is known not only as a pro-lymphangiogenic factor, but also as a lymphatic metastasis-related factor^26,27^. PDPN overexpressed in cancer cells or fibroblasts of cancerous stroma significantly correlates with lymph node metastasis^28–30^. PDGF-BB, which is homo dimmer of PDGFB protein, induces intratumoral lymphangiogenesis and promotes lymphatic metastasis^31–33^. Expression of PDGFB by cancer cells and that of PDGFRβ by tumor-associated stromal cells are strongly associated with lymphatic metastasis^34,35^. CCL21 is expressed in secondary lymphoid organs, binds to CCR7 and facilitates lymphatic invasion of tumor cells^37,38^. On the other hand, in the current study, the gene expression of SPHK1 had no positive correlation with LYVE1 or PROX1, both of which specifically express on the lymphatic endothelial cells and are known as lymphangiogenesis-related factors^36^. In addition, immunohistochemical examination revealed that pSPHK1 expression had no association with LVD in gallbladder cancer (Supplementary Fig. 7a,b). Our data and these reports suggest that SPHK1 in BTC has low contribution to lymphangiogenesis, but promotes lymphatic metastasis, and contributes to the aggressive nature of BTC.

Some demonstrated that SPHK1 expression is associated with lymph node metastasis in human cancer by immunohistochemistry^46–51^. However, most of them examined non-phosphorylated SPHK1 expression, which is not an activated form, and thus may not accurately reflect the production of S1P. Indeed, we previously demonstrated that breast cancer with lymph node metastasis had higher pSPHK1 expression than that without^17^. In the current study, we demonstrated that high expression of pSPHK1 was associated with lymph node metastasis in gallbladder cancer. These results indicate that, like breast cancer, BTC utilizes pSPHK1 to promote lymph node metastasis. We cannot help but speculate that pSPHK1 targeted therapeutics may prevent lymph node metastasis and improve survival of BTC in the future.

Compared to SPHK1, much less is known about SPHK2. Both SPHK1 and SPHK2 produce S1P. However, they show different tissue distribution, subcellular location, and kinetic properties, have unique function, and occasionally play opposite roles^12,52,53^. SPHK1 is localized in the cytosol near the cell membrane, while SPHK2 is located in the organelles such as the nucleus and mitochondria^12,52,53^. SPHK1-produced S1P promotes cell growth and survival, whereas SPHK2 induces pro-apoptotic signals^12,52,53^. S1P produced by SPHK1, not SPHK2, is exported out of cancer cells through transporters, and act in paracrine and autocrine manner, which is known as “inside-out signaling”^12^. Previously we reported that S1P produced by SPHK1, but not SPHK2, is a critical mediator of cancer-induced lymphangiogenesis^16^. In the current study, the gene expression of SPHK2 was not clustered with that of lymphatic metastasis-related factors in the correlation matrix analysis (Fig. 4a). Furthermore, the gene expression of SPHK2 did not positively correlate with that of lymphatic metastasis-related factors in BTC (Supplementary Fig. 6). These data are in agreement with the notion that, in contrast to SPHK1, SPHK2 has low contribution to lymphatic metastasis in BTC.

There are limitations of the current study. First, this study is a retrospective analysis of a limited number of samples or patients. Second, we were not able to analyze the correlation between the amount of sphingolipids and gene expression because we utilized TCGA cohort to analyze the correlation of each gene expression. Third, the contribution of activated SPHK2 to lymph node metastasis was not possible to be assessed by immunohistochemistry in BTC because there is no anti-human phospho-SPHK2 antibody with reliable and widely approved quality. However, this is one of the largest studies describing with the quantified mass levels of sphingolipids and the protein level of pSPHK1 from human BTC tissues. Further studies with larger cohorts may elucidate the association between sphingolipid levels and clinicopathological parameters or survival.

In conclusion, we found that S1P is elevated in BTC with the activation of ceramide-synthetic pathways. Factors that are related to lymphatic metastasis, as well as the ABCC1 transporter, correlated with SPHK1 expression in BTC, which suggests that S1P is exported via ABCC1 and contributes to lymphatic metastasis. Since BTC with lymph node metastasis have significantly higher expression of activated SPHK1, it is speculated that generated S1P contributes to lymphatic metastasis.

## Material and Methods

### Tissue samples

From January 2014 to June 2017, BTC tissues were collected from 15 patients who underwent curative surgical resection for BTC larger than 1.0 cm (gallbladder cancer, n = 5; intrahepatic bile duct cancer, n = 2; and extrahepatic bile duct cancer, n = 8). Normal biliary tract tissues were collected from 17 patients (gallbladder mucosa, n = 5; and extrahepatic bile duct mucosa, n = 12), 13 cases of which were the matched cases (gallbladder cancer, n = 5; and extrahepatic bile duct cancer, n = 8), and 4 of which were the unmatched cases (ampullary cancer, n = 3; and duodenal cancer, n = 1). All tissues were snap-frozen and stored at −80 °C. All the patients agreed to provide the tissue for this study with informed consent. This study protocol was approved by the Institutional Review Board of Niigata University Medical and Dental Hospital. The informed consent was obtained from all patients. All methods in this study were performed in accordance with the relevant guidelines and regulations, including the 1975 Declaration of Helsinki. For immunohistochemical examination, we collected gallbladder cancer tissues from 20 patients who underwent curative surgical resection, 10 cases with lymph node metastasis, the others without lymph node metastasis in the pathological diagnosis. All 20 cases had invasion to the subserosa.

### Quantitation of sphingolipids by liquid chromatography-electrospray ionization-tandem mass spectrometry (LC-ESI-MS/MS)

Lipids were extracted from tissue samples at Virginia Commonwealth University Lipidomics Core as follows; Samples were collected into 13 × 100 mm borosilicate tubes with a Teflon-lined cap (VWR, West Chester, PA). Then 2 mL of CH_3_OH and 1 mL of CHCl_3_ were added along with the internal standard cocktail (250 pmol of each species dissolved in a final total volume of 20 µL of ethanol, methanol, and water which are mixed in a ratio of 7:2:1). After sonication for 30 seconds, this mixture was incubated at 48 °C overnight. After cooling down, 150 µL of 1 M KOH in CH_3_OH was added and, after brief sonication, incubated in a shaking water bath for 2 hours at 37 °C to hydrolyze glycerolipids. The extract was brought to neutral pH with 12 µL of glacial acetic acid, transferred to a new tube, and reduced to dryness using a Speed Vac. The dried residue was reconstituted in 0.5 mL of the starting mobile phase solvent for LC-MS/MS analysis, sonicated for 15 seconds, then centrifuged for 5 minutes in a tabletop centrifuge before transfer of the clear supernatant to the autoinjector vial for analysis. Then, sphingolipids were quantified by LC-ESI-MS/MS (4000 QTRAP, ABI) at Virginia Commonwealth University Lipidomics Core as described previously^13,16,54^.

### Pathologic examination

All of the surgically resected specimens were submitted to the Department of Surgical Pathology of Niigata University Medical and Dental Hospital and examined by two experienced pathologists who lacked access to the clinical data. Paraffin-embedded blocks from each resected specimen were used for immunohistochemistry. Five serial 4-μm sections were recut and used for staining with hematoxylin and eosin, pSPHK1, PDPN (D2-40) and negative controls.

pSPHK1 stain was performed as we previously reported^17,18^. Briefly, antigen retrieval for pSPHK1 was performed by microwaving the slides under pressure in a citrate buffer for 10 min (pH 9.0). Endogenous peroxidase was blocked using 0.3% hydrogen peroxide for 20 min. After blocking nonspecific background, the sections were incubated overnight with the primary antibody (SPHK1 polyclonal antibody; 1:100 dilution; ECM Biosciences LLC, Versailles, KY) at 4 °C. Then, the sections were incubated with biotinylated rabbit antimouse streptavidin-peroxidase complex for 10 min. Diaminobenzidine was used as the chromogen, and the sections were counterstained with hematoxylin. Normal mouse immunoglobulin was substituted as the primary antibodies in the negative control. The vascular and lymphatic endothelial cells of all vessels reacted with the antibody against pSPHK1. As a result, the pSPHK1 staining intensity of endothelial cells was considered moderate staining, and the pSPHK1-staining intensity of BTC cells was registered as follows: 0 (negative), 1 (weak), 2 (moderate), or 3 (strong), based on comparison with endothelial cell staining. The extent of stained cells was registered as follows: 0 (0–25%), 1 (26–50%), 2 (51–75%), 3 (76–100%). The final immunoreactive score was assessed by multiplying the intensity and extent score, with the minimum score of 0 and the maximum score of 12. The staining score of 0 to 5 was considered as pSPHK1-negative, and 6 to 12 considered as pSPHK1-positive.

To retrieve antigen for D2-40, the sections were autoclaved at 120 °C for 20 minutes in a citrate buffer (pH 6.0). Endogenous peroxidase was blocked using 0.3% hydrogen peroxide for 20 min. After blocking nonspecific background, the sections were incubated overnight at 4 °C with mouse anti-D2-40 monoclonal antibody (prediluted, Nichirei Biosciences, Japan). The sections were incubated with biotinylated rabbit antimouse streptavidin-peroxidase complex for 10 min. Diaminobenzidine was used as the chromogen, and the sections were counterstained with hematoxylin. As a negative control, normal mouse immunoglobulin was substituted for the primary antibody. D2-40-stained sections were scanned at low magnification (×10 eyepiece, ×10 objective) for assessing intratumoral LVD. After detecting the areas with the greatest number of discrete lymphatic vessel staining, referred to as “hot spots”, we quantified intratumoral LVD by counting the number of lymphatic vessels in 1 mm^2^ “hot spots”. The LVD of 0–7 was considered as low, and more than 7 considered as high. The cut off value was set according to the median number of LVD.

### Data acquisition and pre-processing using TCGA cohort

TCGA is a project co-managed by the National Cancer Institute and the National Human Genome Research Institute of the National Institute of Health which has collected primary cancer samples from over 10,000 patients on over 30 tumor types to catalogue genomic and epigenomic data for cancer using high-throughput sequencing techniques. There are 36 and 9 samples of cancer and non-cancer tissue in cholangiocarcinoma cohort (CHOL) of TCGA. The gene expression level quantification data (mRNA expression z-score from RNA-seq) for the TCGA CHOL cohort were downloaded through cBioPortal (http://www.cbioportal.org/) and UCSC Genome Browser (http://genome.ucsc.edu/) and used as previously described^22,55–58^.

### Statistical analysis

Except for TCGA analysis, all statistical evaluations were performed using the SPSS 23.0 J software package (SPSS Japan, Tokyo, Japan). Continuous variables between two groups were compared by the Mann-Whitney U test, and categorical variables between two groups were compared by the Fisher’s exact test. In TCGA analysis, Pearson correlations were calculated based on the expression levels of interested genes in cancer tissues and plotted. The hierarchical clustering method was used for the grouping of the genes. All TCGA statistical analyses were performed using R software (http://www.r-project.org/) and Bioconductor (http://bioconductor.org/). All tests were two-sided, and *P* values < 0.05 were considered statistically significant.

## Electronic supplementary material

Supplementary Figures 1-7
