# Supplementary material for: Generation of sphingosine-1-phosphate is enhanced in biliary tract cancer patients and is associated with lymphatic metastasis
Source: Sci Rep. 2018 Jul 17;8:10814. doi: 10.1038/s41598-018-29144-9 (PMC6050292; doi:10.1038/s41598-018-29144-9)
Supplement: Supplementary file 1 — Supplementary Figures 1-7 [file 41598_2018_29144_MOESM1_ESM.pdf]

Title:

Generation of sphingosine-1-phosphate is enhanced in biliary tract cancer patients and is associated with lymphatic metastasis

Yuki Hirose[a*], Masayuki Nagahashi[a+], Eriko Katsuta[b*], Kizuki Yuza[a], Kohei Miura[a], Jun Sakata[a], Takashi Kobayashi[a], Hiroshi Ichikawa[a], Yoshifumi Shimada[a], Hitoshi Kameyama[a], Kerry-Ann McDonald[b], Kazuaki Takabe[a-e], Toshifumi Wakai[a]

[a] Division of Digestive and General Surgery, Niigata University Graduate School of Medical and Dental Sciences, Niigata City, Niigata 951-8510, Japan

[b] Breast Surgery, Department of Surgical Oncology, Roswell Park Comprehensive Cancer Center, Buffalo, New York 14263, USA

[c] Department of Surgery, University at Buffalo Jacobs School of Medicine and Biomedical Sciences, the State University of New York, Buffalo, New York 14203, USA

[d] Department of Breast Surgery and Oncology, Tokyo Medical University, Shinjuku-ku, Tokyo 160-8402, Japan.

[e] Department of Surgery, Yokohama City University, Kanazawa-ku, Yokohama 236-0004, Japan.

* These authors contributed equally to this study

[+]**Corresponding author:**

Masayuki Nagahashi, M.D., Ph.D.

Division of Digestive and General Surgery

Niigata University Graduate School of Medical and Dental Sciences

1-757 Asahimachi-dori, Chuo-ku, Niigata City, 951-8510 Japan

E-mail: mnagahashi@med.niigata-u.ac.jp

Telephone: +81-25-227-2228

Fax: +81-25-227-0779

Supplementary Figure 1

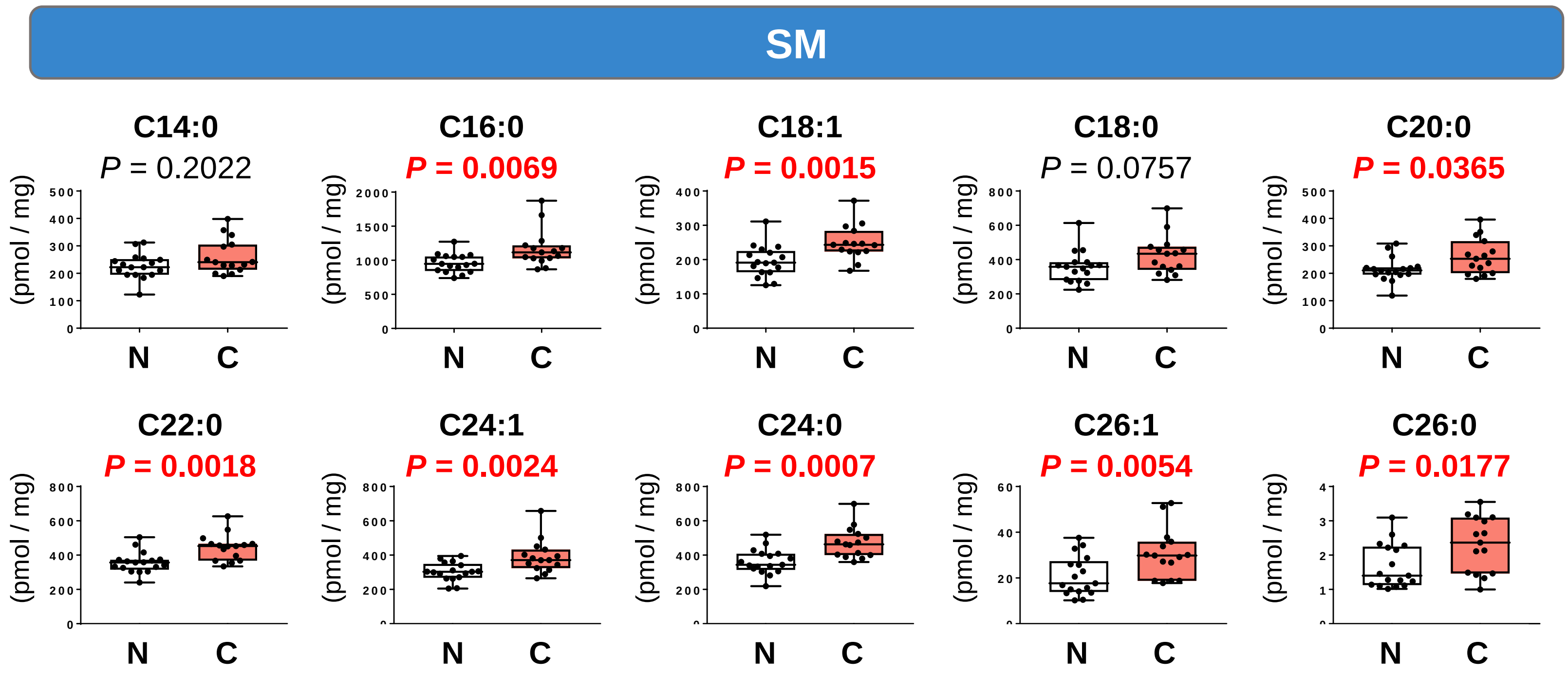

Supplementary Figure 1. The level of SM species (C14:0, C16:0, C18:1, C18:0, C20:0, C22:0, C24:1, C24:0, C26:1, and C26:0) in biliary tract cancer (n = 15) and normal biliary tract tissue (n = 17) were determined by LC-ESI-MS/MS. Mean values are shown by the horizontal lines. Two-sided *P* values < 0.05 were considered statistically significant (Mann-Whitney U test). N: normal biliary tract tissue; C: biliary tract cancer tissue.

Supplementary Figure 2

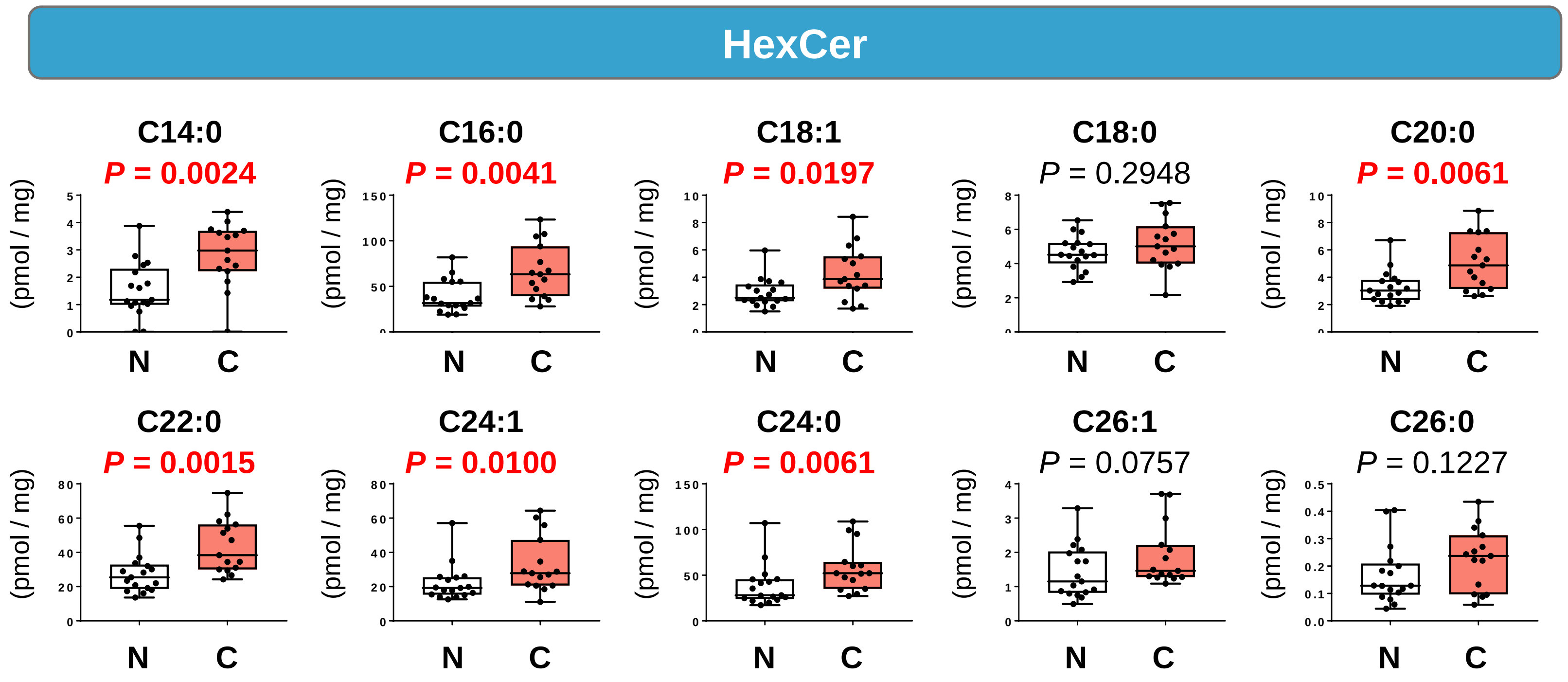

Supplementary Figure 2. The level of HexCer species (C14:0, C16:0, C18:1, C18:0, C20:0, C22:0, C24:1, C24:0, C26:1, and C26:0) in biliary tract cancer (n = 15) and normal biliary tract tissue (n = 17) were determined by LC-ESI-MS/MS. Mean values are shown by the horizontal lines. Two-sided *P* values < 0.05 were considered statistically significant (Mann-Whitney U test). N: normal biliary tract tissue; C: biliary tract cancer tissue.

Supplementary Figure 3

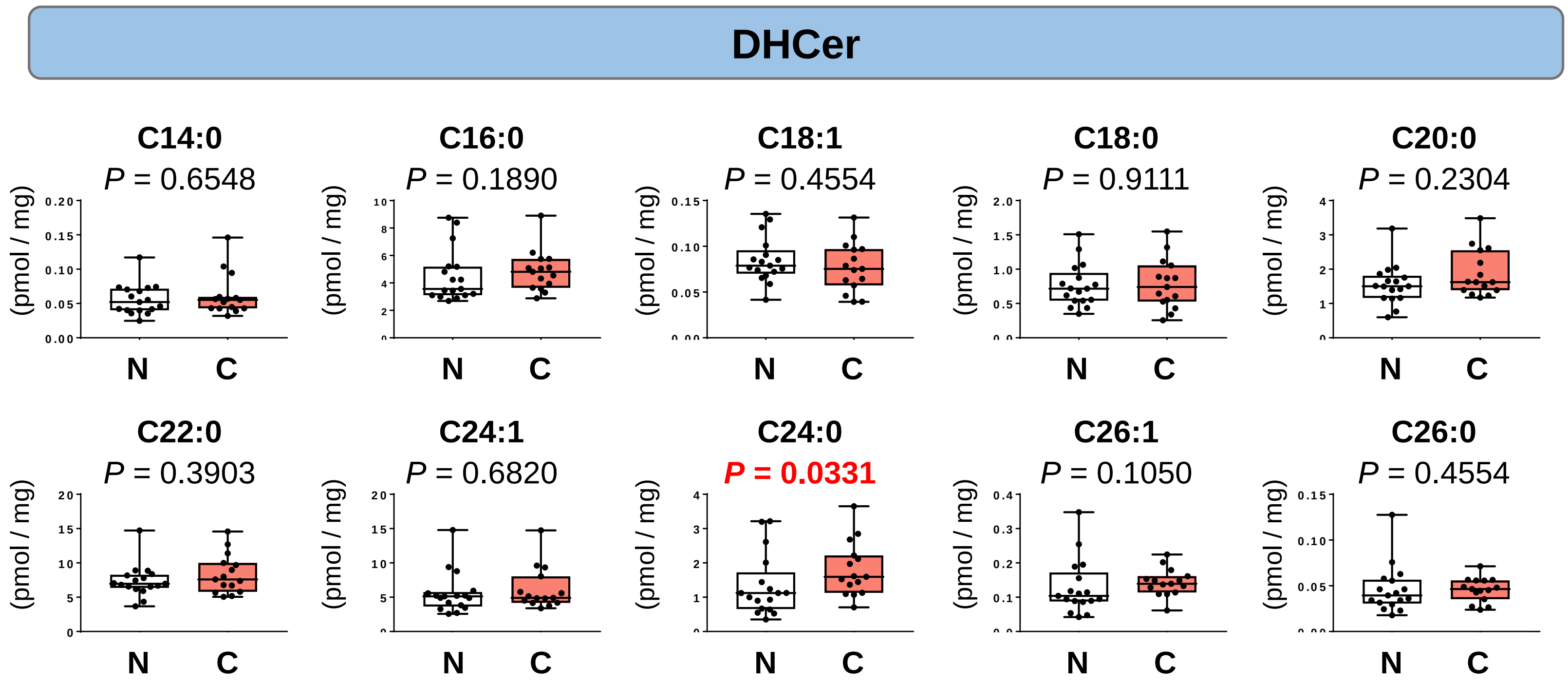

Supplementary Figure 3. The level of DHCer species (C14:0, C16:0, C18:1, C18:0, C20:0, C22:0, C24:1, C24:0, C26:1, and C26:0) in biliary tract cancer (n = 15) and normal biliary tract tissue (n = 17) were determined by LC-ESI-MS/MS. Mean values are shown by the horizontal lines. Two-sided *P* values < 0.05 were considered statistically significant (Mann-Whitney U test). N: normal biliary tract tissue; C: biliary tract cancer tissue.

Supplementary Figure 4

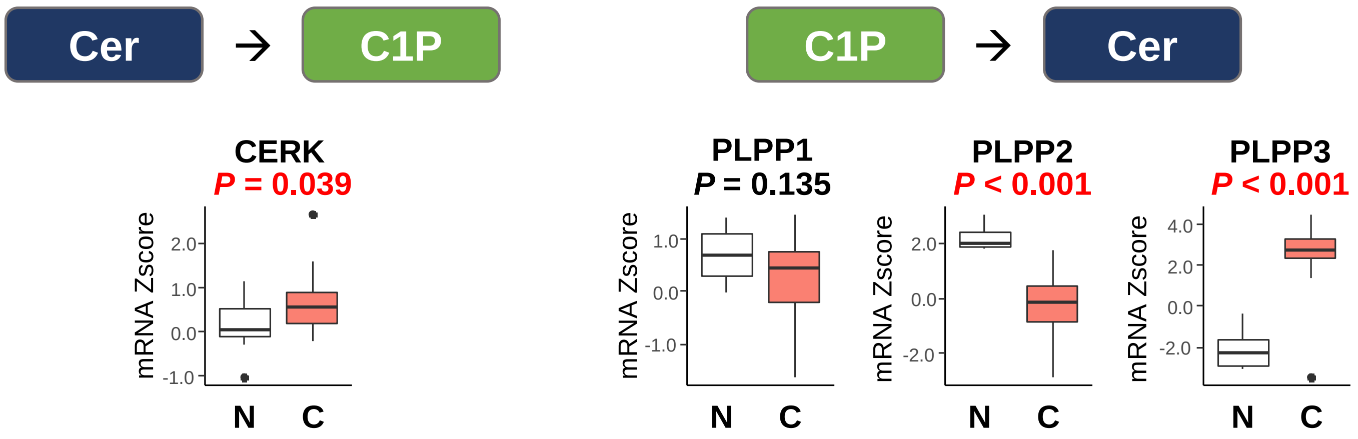

Supplementary Figure 4. The gene expression level of CERK and PLPPs in TCGA cohort (36 and 9 samples of cancer and non-cancer tissue in cholangiocarcinoma cohort) were analyzed. Mean values are shown by the horizontal lines. Two-sided *P* values < 0.05 were considered statistically significant (Mann-Whitney U test). N: normal biliary tract tissue; C: biliary tract cancer tissue.

Supplementary Figure 5

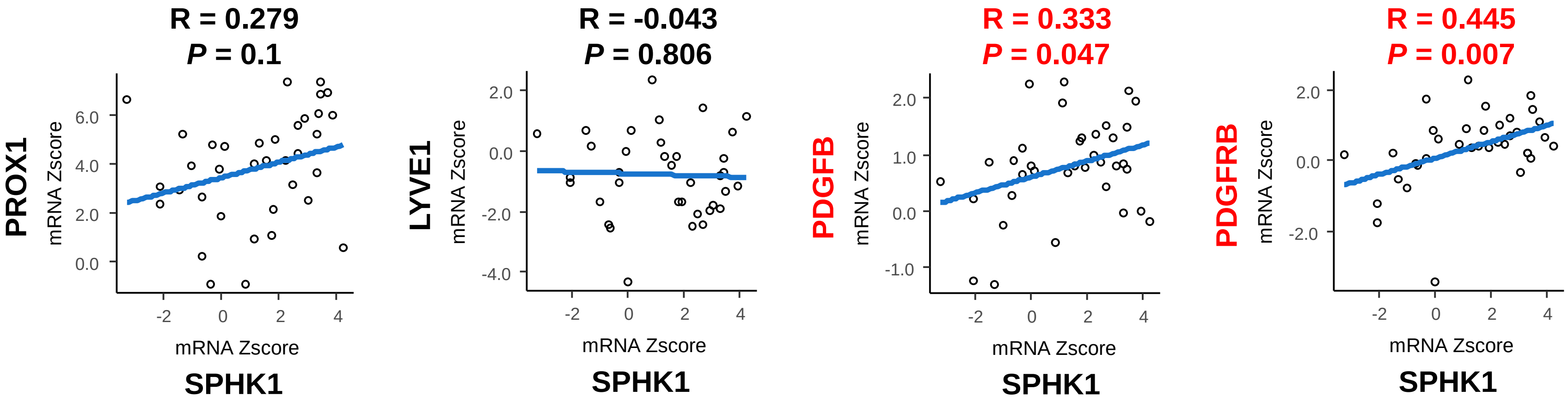

Supplementary Figure 5. Pearson correlations were calculated between gene expression level of SPHK1 and those of PROX1, LYVE1, PDGFB, and PDGFRβ in biliary tract cancer in TCGA cohort (36 samples of cancer tissue in cholangiocarcinoma cohort). All tests were two-sided, and *P* values < 0.05 were considered statistically significant.

Supplementary Figure 6

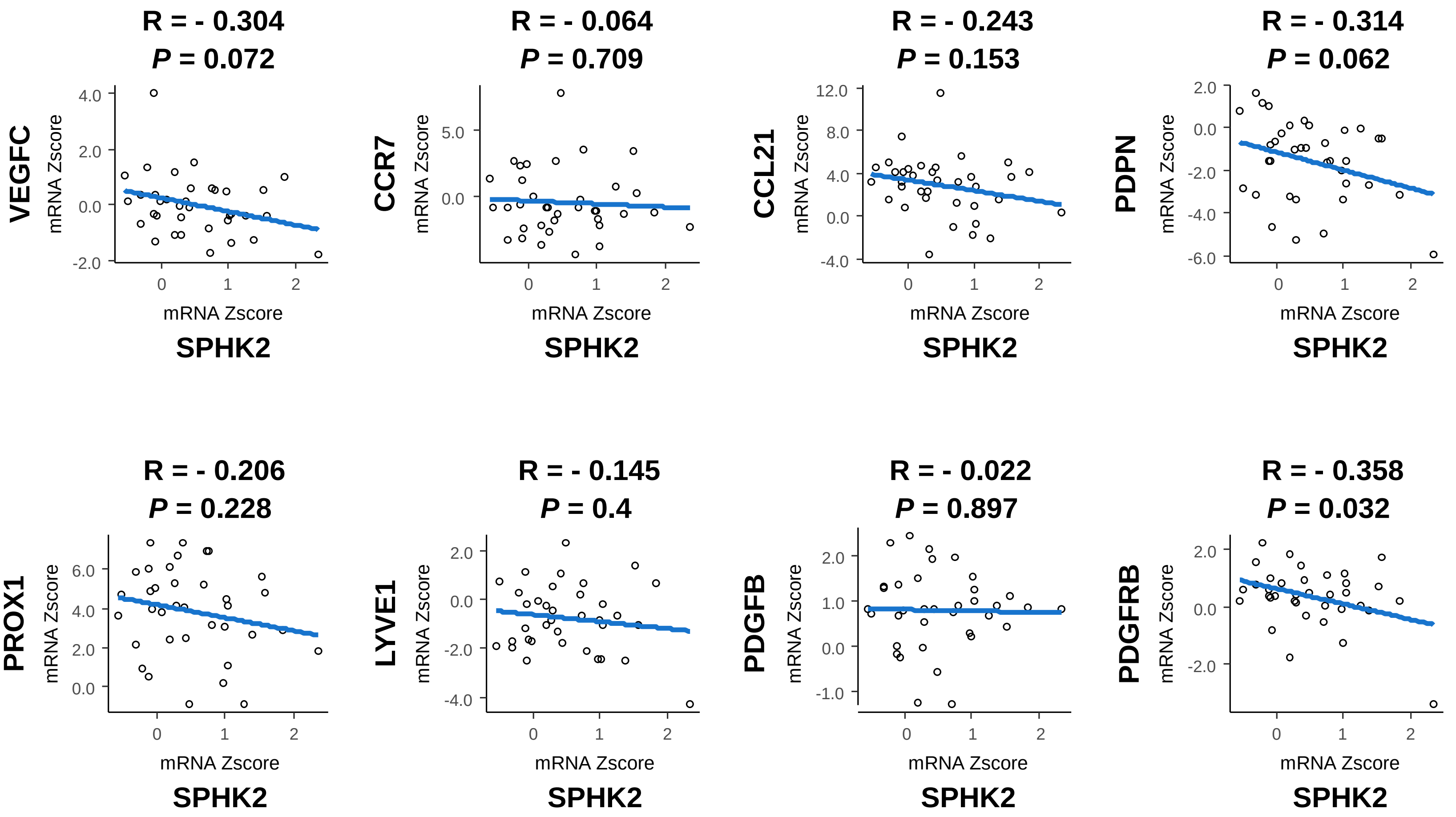

Supplementary Figure 6. Pearson correlations were calculated between gene expression level of SPHK2 and those of VEGFC, CCR7, CCL21, PDPN, PROX1, LYVE1, PDGFB, and PDGFRβ in biliary tract cancer in TCGA cohort (36 samples of cancer tissue in cholangiocarcinoma cohort). All tests were two-sided, and *P* values < 0.05 were considered statistically significant.

Supplementary Figure 7

a

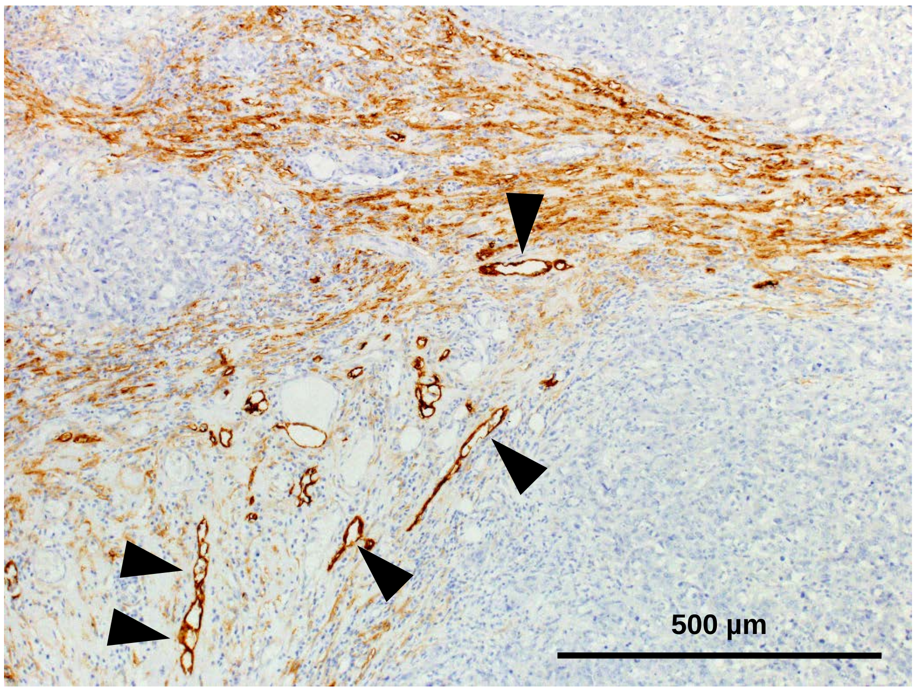

b

	No. of patients with		
	pSPHK1 high expression	pSPHK1 low expression	*P* value
Intratumoral lymphatic vessel density			> 0.999
High	6	2	
Low	9	3	

Supplementary Figure 7. (a) Immunohistochemical staining of D2-40 in biliary tract cancer is shown. Arrow heads shows lymphatic vessels in gallbladder cancer. Scale bar = 500 μm. (b) The association between the pSPHK1 expression and intratumoral lymphatic vessel density in gallbladder cancer tissues with (n = 10) or without lymph node metastasis (n = 10) was evaluated by the Fisher's exact test. All tests were two-sided, and *P* values < 0.05 were considered statistically significant.
